# Case of Hydrophobia, in Which Blood-Letting Was Unsuccessful

**Published:** 1815-01

**Authors:** R. Bellingham

**Affiliations:** Bourn, Lancashire


*For the Medical and Physical Journal.*

### *Case of Hydrophobia, in which Blood-letting was Unsuccessful;* by Mr. R. Bellingham.

THE attention and the hopes of the medical world have been of late so strongly excited by the publications of Dr. Shoolbred on the subject of Hydrophobia, that it would be a positive dereliction of duty to withhold the communication of the following case of that terrible and intractable disease. It would have afforded, however, much more satisfaction faction to have been able to support the practice which Dr. S. has recommended, by the publication of an instance of its successful employment, than it can do to lessen its claims to our confidence by an example of its failure. As far as a single case is to be dwelt upon, (and happily an accumulation of facts upon this subject never falls within the practice of any one,) this case deserves to be recorded, inasmuch as it will appear that the system of depletion had the fairest and the fullest trial.

William Percival, aged three years and a half, a child possessing unusual quickness and intelligence, was bitten on the 13th of October, in the inside of the upper lip, by a dog, who had betrayed no previous symptoms of madness, though suspected of having been bitten some time before by another dog in a rabid state. The child was immediately brought by his father to Mr. Holland, surgeon, at Market Deeping, under whose care he afterwards remained. In conjunction with Mr. Holland, I attended the patient in the sequel, and at his request and with his assistance I made minutes of the case, and now present them to the public. Being furnished by him with the particulars antecedent to the accession of the hydrophobic symptoms, as the wound was slight and superficial, being rather an abrasion of the delicate cuticle of the part, than a laceration of its substance; and as there was no proof of the rabid state of the animal by whom it was inflicted, Mr. H., supported by the opinion of another surgeon, who happened to be present, applied the potassa fusa freely to the part, so as to produce extensive sloughing. This application was occasionally repeated during the ensuing month, so as to occasion successive sloughs, and the child remained in perfect health to the 16th of November.

About eight o'clock on the morning of the 17th, the father came to Mr. H. in great alarm for the safety of his child. At nine o'clock, when he first visited him, we had the following account: that the child had betrayed some marks of indisposition the evening before, had been fretful, complained of headache, which was attributed to a fall he received the day before, by which one eye was considerably bruised; that he passed a restless night, with general symptoms of fever; but no particular alarm was excited till about seven o'clock, when, in attempting to take some tea, he was observed to have some difficulty in deglutition, and to attempt it with much reluctance. The symptoms were now as follow: the child appeared dull and listless, the eyes were heavy, the pulse small and about 120 in the minute, the tongue moist but covered with a brownish fur, the bowels regular.

regular. When questioned as to the seat of pain, he pointed constantly to the throat, about the thyroid cartilage, and occasionally to the head. On being offered a little gruel, he refused; but, with some persuasion, and not without some effort, he swallowed about a spoonful; and evidently, both before and at the moment of swallowing, with a spasmodic action of the muscles of deglutition, though not severe. A vein of the arm was opened, and suffered to bleed by a free orifice *ad deliquium;* the quantity taken away was eleven ounces; the pulse immediately sunk to 60; a warm bath was ordered to be in readiness, but the prostration remained so great that its employment was postponed.

℞ Hydr. Submur. Pulv. Antim. āā gr. j.
Pulv. Jalapæ gr. ij. Misce fiat pulvis 2da.
quâque horâ sumendus.

Half past three, p. m. our visit was repeated. He had taken four powders without effect; had constantly refused either food or drink; the spasms had but little increased in frequency or violence; pulse 140, and fuller; the blood exhibited no inflammatory appearances. A bason of water being brought, the patient was desired to immerse his hand: this he attempted, but shrunk back before it touched the water with much agitation. On his hand being forced into it, the organs of deglutition were evidently much affected; the wounded part shewed no altered or unnatural appearance. No evacuation having been procured, enemas were ordered to be injected, containing Magnes. Sulphatis et Ol. Ricini āā ℥ſs. Continuentur Pulveres.

Half past twelve (midnight).—Had been very restless for about three hours, but was now quiet; the attendants had more than once thought him expiring; pulse 144, and much fuller. Some water was shewn to him in a glass, from which he was urged to drink, but as usual refused; the agitation of it with a spoon, though removed entirely from his sight, evidently affected him. The powders had not yet acted, and the enemas had been neglected, but one was now immediately employed. The bandage was removed from the arm, and eight ounces of blood taken away: this flowed with the greatest force and freedom, without the application of any ligature above the orifice in the vein. The arterial action at this time seemed to be very great. He still referred his uneasiness entirely to the throat and head, but seemed perfectly easy about the stomach, and evinced no tenderness on pressure, though upon this point he was repeatedly interrogated, and examined by the hand. After this bleeding he was for the space of a minute violently and univer-

3 sally sally convulsed, but, on being returned to his bed, expressed himself perfectly free from pain.

℞ Hydr. Submur. Pulv. Antim. āā gr. j.
Pulv. Opii gr. iii. Misce fiat pulvis tertia quâque horâ sumendus.

Pulse at the time of leaving him 120. The glyster to be repeated in two hours, if required. The urine was pale in colour and deposited a whitish sediment. The glyster operated briskly in less than an hour, and he remained more tranquil till five o'clock, when the spasms became more violent and general, and returned in more rapid succession till nine, when, in a severe convulsive struggle, he expired. Two of the powders had been swallowed with great eagerness, though with increased difficulty, but without procuring any rest. His senses remained perfect to the last moment. No discharge of saliva was observed, nor were the organs of respiration particularly affected.

Dr. Skrimshire, of Peterborough, was sent for, but his arrival was too late. By him, as well as subsequently by Mr. Holland and myself, every perstuasive argument was used, in order to obtain permission to inspect the body; but the prejudices of the friends could not possibly be surmounted, and I very much regret that in this respect the narration of the case is incomplete. How far the appearances on dissection would have tended in this case to establish or refute the identity, or at least the analogy, between this disease and Gastritis, it is impossible to determine. In the rapid progress of the case, however, this point was not lost sight of; and, as far as the most attentive survey of the symptoms would enable me to form a conclusion, that conclusion would be adverse to such a supposition, and for the following reasons:—1st. There was never any reference of pain to the region of the stomach, though repeatedly questioned upon that point. 2dly. There was no tenderness on pressure, though examined in the most cautious manner. 3dly. The fluids and medicines which he swallowed were never rejected, nor was there even a disposition to sickness. 4thly. There was not that peculiar cast of features, that look of anxiety and anguish, which characterises Gastritis; his countenance had more of listlessness than anxiety. I have omitted to mention in its proper place, that the second portion of blood shewed no inflammatory characters, though its crassamentum had decidedly more tenacity than the first. It is worthy of remark, that after the first copious bleeding, for I consider the detraction of eleven ounces from a patient of that age as copious, the pulse increased considerably both in frequency and strength. The temperature of the skin was never much beyond its proper standard. Every one who takes the trouble of perusing this case, will of course form his own conclusions; nor would I presume, or even wish, to direct or influence them; for myself I confess, though I should be far from sanguine as to the issue, should a similar case occur within my observation, yet with the same increased action of the arterial system, the same marks of a tendency to inflammation, I should still be disposed to say, "Melius est anceps remedium experiri quam nullum." Of the powers of mercury, either prophylactic or curative, I should entertain much doubt; I should even suspect it of adding to rather than allaying the morbid irritability of the system; but it may, I think, be made a question, whether the liberal employment of opium, or some other powerful narcotic, might not be compatible with the vigorous use of the lancet.

*Bourn, Lancashire,* R. BELLINGHAM.

*Nov.* 27, 1814.

